# Effect of a High-Fat Diet on the Small-Intestinal Environment and Mucosal Integrity in the Gut-Liver Axis

**DOI:** 10.3390/cells10113168

**Published:** 2021-11-14

**Authors:** Takashi Nakanishi, Hirokazu Fukui, Xuan Wang, Shin Nishiumi, Haruka Yokota, Yutaka Makizaki, Yoshiki Tanaka, Hiroshi Ohno, Toshihiko Tomita, Tadayuki Oshima, Hiroto Miwa

**Affiliations:** 1 Division of Gastroenterology and Hepatology, Department of Internal Medicine, Hyogo College of Medicine, Nishinomiya 663-8501, Japan; ta-nakanishi@hyo-med.ac.jp (T.N.); xwang31@tmu.edu.cn (X.W.); tomita@hyo-med.ac.jp (T.T.); t-oshima@hyo-med.ac.jp (T.O.); miwahgi@hyo-med.ac.jp (H.M.); 2 Department of Omics Medicine, Hyogo College of Medicine, Nishinomiya 663-8501, Japan; sh-nishiumi@hyo-med.ac.jp; 3 R&D Center, Biofermin Pharmaceutical Co., Ltd., Kobe 651-2242, Japan; yokota_haruka@biofermin.co.jp (H.Y.); makizaki_yutaka@biofermin.co.jp (Y.M.); tanaka_yoshiki@biofermin.co.jp (Y.T.); ohno_hiroshi@biofermin.co.jp (H.O.)

**Keywords:** high-fat diet, small intestine, microbiome, bile acid, barrier

## Abstract

Although high-fat diet (HFD)-related dysbiosis is involved in the development of steatohepatitis, its pathophysiology especially in the small intestine remains unclear. We comprehensively investigated not only the liver pathology but also the microbiome profile, mucosal integrity and luminal environment in the small intestine of mice with HFD-induced obesity. C57BL/6J mice were fed either a normal diet or an HFD, and their small-intestinal contents were subjected to microbial 16S rDNA analysis. Intestinal mucosal permeability was evaluated by FITC-dextran assay. The levels of bile acids in the small-intestinal contents were measured by liquid chromatography/mass spectrometry. The expression of tight junction molecules, antimicrobial peptides, lipopolysaccharide and macrophage marker F4/80 in the small intestine and/or liver was examined by real-time RT-PCR and immunohistochemistry. The abundance of *Lactobacillus* was markedly increased and that of *Clostridium* was drastically decreased in the small intestine of mice fed the HFD. The level of conjugated taurocholic acid was significantly increased and those of deconjugated cholic acid/secondary bile acids were conversely decreased in the small-intestinal contents. The expression of occludin, antimicrobial Reg IIIβ/γ and IL-22 was significantly decreased in the small intestine of HFD-fed mice, and the intestinal permeability was significantly accelerated. Infiltration of lipopolysaccharide was significantly increased in not only the small-intestinal mucosa but also the liver of HFD-fed mice, and fat drops were apparently accumulated in the liver. Pathophysiological alteration of the luminal environment in the small intestine resulting from a HFD is closely associated with minimal inflammation involving the gut-liver axis through disturbance of small-intestinal mucosal integrity.

## 1. Introduction

Accumulating evidence has revealed that the gut microbiome plays pivotal roles in the pathophysiology of various diseases, such as inflammatory disorders [1], metabolic syndromes or psychological disorders affecting the whole body [2]. Indeed, the gut microbiome is a key player in the pathophysiology of the gut-liver axis [3]. For instance, alteration of the gut microbiome profile affects the intestinal mucosal barrier function and/or the immune system [4], triggering inflammatory conditions in not only the intestinal mucosa but also in the liver via the portal vein [5].

The diet is a crucial factor determining the profile of the gut microbiome [6]. It has been reported that a high-fat diet (HFD) is a potent inducer of gut microbiota imbalance (dysbiosis) and responsible for the development of metabolic diseases (obesity or diabetes) [7]. However, it still remains unclear how HFD-related dysbiosis is involved in dysfunction of the intestinal mucosal barrier and/or steatohepatitis. On the other hand, although the small intestine is a critically important organ for lipid absorption and a barrier against bacterial translocation into the liver, little is known about HFD-associated pathophysiology in the small intestine. Therefore, to clarify the role of HFD-related dysbiosis in the intestinal mucosal barrier and/or steatohepatitis, we comprehensively investigated not only the liver pathology but also the microbiome profile, mucosal integrity and luminal environment in the small intestine of mice with HFD-induced obesity.

## 2. Materials and Methods

### 2.1. Animal Model

Specific pathogen-free mice (C57BL/6J, five weeks old, male) were obtained from Japan SLC (Shizuoka, Japan), housed at 22 ± 3 °C under a 12:12-h light-dark cycle and used for the following experiments. All experimental procedure were approved by the Animal Use and Care Committee of Hyogo College of Medicine. In addition, all experiments described below were performed in accordance with relevant guidelines and regulations.

After a one-week adaptation period, the mice were fed either a normal diet (D12450J; Research Diet, New Brunswick, NJ, USA) or an HFD (D12492; Research Diet) for eight weeks. The diet compositions are shown in Supplementary Table S1. The body weight of the experimental mice was recorded weekly. After removal, the content of the whole small intestine was collected in tubes and immediately snap-frozen in liquid nitrogen and stored at −80 °C until use. The removed small-intestinal tissues were divided into the jejunum and the ileum, cut open along the longitudinal axis, and fixed in neutral aqueous phosphate-buffered 10% formalin for histological examinations, or stored in nitrogen liquid for real-time RT-PCR.

### 2.2. Real-Time RT-PCR

Total RNA was isolated from the small-intestinal tissues with TRIzol reagent (Invitrogen, Waltham, MA, USA). Four micrograms of total RNA was reverse-transcribed using an oligo (dT) primer (Applied Biosystems, Branchburg, NJ, USA), and real-time RT-PCR was carried out using a 7900H Fast Real-Time PCR System (Applied Biosystems) as previously described [8]. The set of primers used is shown in Supplementary Table S2. Real-time RT-PCR assays were carried out with 200 ng of RNA-equivalent cDNA, SYBR Green Master Mix (Applied Biosystems) and 500 nmol/l gene-specific primers. The PCR cycling conditions were 50 °C for 15 s and 60 °C for 60 s. The intensity of the fluorescent dye was determined, and the expression levels of target gene mRNAs were normalized to those of *glyceraldehyde-3-phosphate dehydrogenase* (*GAPDH*) mRNA.

### 2.3. Immunohistochemistry and Nile Blue Staining

Immunohistochemical staining was performed with an Envision Kit (Dako Agilent Technologies, Tokyo, Japan) in accordance with the manufacturer’s protocol [9] using anti-lipopolysaccharide (LPS) (dilution 1: 50,000; Abcam, Cambridge, UK), anti-lysozyme (dilution 1: 1000; Abcam, Cambridge, UK) and anti-CD3 antibodies (dilution 1: 50; Abcam, Cambridge, UK).

Immunohistochemical double-staining was performed as previously described [10]. In brief, the small-intestinal and liver sections were incubated with a mouse anti-LPS antibody (dilution 1:1000, Abcam), anti-F4/80-eFluor 570-labeded antibody (dilution 1:100, Invitrogen, Camarillo, CA, USA) or anti-occludin antibody (dilution 1:25, Invitrogen, Camarillo, CA, USA) for 60 min at room temperature. The sections were then incubated with fluorescein isothiocyanate-labeled anti-mouse immunoglobulin (1: 1000; Dako Agilent Technologies, Tokyo, Japan) or tetramethyl rhodamine isothiocyanate-labeled anti-rabbit immunoglobulin (1: 1000; Dako Agilent Technologies, Tokyo, Japan) for 30 min at room temperature. After washing in phosphate-buffered saline, the sections were counterstained with Antifade mountant with DAPI (Life Technologies, Carlsbad, CA, USA). Images were acquired and analyzed using a Zeiss LSM 780 confocal microscope (Carl Zeiss Japan, Tokyo, Japan).

Liver tissues were fixed in 10% neutral buffered formalin and immersed in gradient sucrose solution (15–30%). Frozen sections were cut and incubated in Nile blue solution (Muto Pure Chemical, Tokyo, Japan) at 60 °C for 20 min, followed by incubation in 1% acetic acid solution.

The positive cells in the small-intestinal mucosa were evaluated as previously reported [8]. The positive cells in the liver were counted in at least four different intralobular areas in a tissue section, and an average was calculated for each mouse.

### 2.4. Illumina Library Generation and DNA Sequencing

Extraction of bacterial DNA was performed as previously described [11,12]. In brief, the small-intestinal contents were washed with PBS and then resuspended in a mixture of 450 μL of extraction buffer and 50 μL of 10% sodium dodecyl sulfate. Then, 300 mg of glass beads and 500 μL of buffer-saturated phenol were added to the solution, and the cells were disrupted with Micro Smash (4000 rpm, 10 s, TOMY SEIKO, Tokyo, Japan), centrifuged (20,000× *g* for 10 min) and 400 μL of the supernatant was collected. The DNA was eluted from the supernatant by the phenol-chloroform method.

Analysis of the 16S rDNA of the small-intestinal contents was performed according to a method previously described [13] (Supplementary Methods S1). In brief, the V3–V4 region of 16S rDNA was amplified using the primers and ligated with overhang Illumina adapter consensus sequences, as previously reported [11]. The PCR was performed on a Veriti thermal cycler (Thermo Fisher Scientific, Waltham, MA, USA), and the amplicon was purified using AMPure XP magnetic beads (Beckman Coulter, Brea, CA, USA). To incorporate two unique indices in the 16S amplicons, PCR reactions were performed as previously described [11,14]. The libraries were purified using AMPure XP beads, quantified fluorometrically using a QuantiT PicoGreen ds DNA Assay Kit (Invitrogen, Paisley, UK), and then pooled for multiplex sequencing. Sequencing was conducted using a 2 × 250-bp paired-end run on a MiSeq platform with MiSeq Reagent Kit v2 chemistry (Illumina).

Demultiplexing and removal of indices were performed using the MiSeq Reporter software (Illumina) as previously reported [15]. Construction of operational taxonomic units (OTUs) and taxonomy assignment were performed using the Quantitative Insights into Microbial Ecology (QIIME) pipeline (http://qiime.org/1.4.0/, accessed on 1 November 2021) [16]. After filtering out the low-quality and/or chimera sequences, 30,000 raw reads were randomly obtained from the sequence files for each sample and merged by fastq-join using the default setting. For each sample, 5000 high-quality sequence reads were randomly obtained, and OTUs for the total high-quality reads were constructed and then assigned to the 16S rRNA gene database using UCLUST with 97% identity. Comparison of each taxon in the gut microbiota was conducted at both the genus and species levels. The Chao1 indices were calculated to examine the alpha diversity of the microbiota in the samples.

### 2.5. Measurement of Bile Acids

The levels of bile acids in the small-intestinal content were measured using liquid chromatography/mass spectrometry (LC/MS). Briefly, the small-intestinal content was homogenized in 1000 μL of methanol containing cholic acid-d4 (d4-CA) (Cayman Chemical Company, MI, USA), chenodeoxycholic acid-d4 (d4-CDCA) (Cayman Chemical Company), taurocholic acid-d5 (d5-TCA) (Toronto Research Chemicals, Inc., North York, Canada), taurochenodeoxycholic acid-d4 (d4-TCDCA) (Cayman Chemical Company), glycocholic acid-d4 (d4-GCA) (Cayman Chemical Company), deoxycholic acid-d4 (d4-DCA) (Cayman Chemical Company), lithocholic acid-d4 (d4-LCA) (Cayman Chemical Company) and glycodeoxycholic acid-d4 (d4-GDCA) (Santa Cruz Biotechnology Inc., CA, USA) as the internal standards. The homogenates were centrifuged at 16,000 × g for 5 min at 4 °C, and then the supernatants were collected and diluted with methanol before LC/MS analysis. LC/MS analysis was performed using a LCMS-8060 (Shimadzu Co., Kyoto, Japan) accompanied by the LC/MS/MS Method Package for Bile Acids (Shimadzu Co.), which contains method information regarding the LC analytical conditions and multiple reaction monitoring parameters used for bile acid analysis. In this study, the LC analytical conditions, which were designed in the LC/MS/MS Method Package for Bile Acids, were changed as follows: One of the mobile phases (mobile phase B) was changed to acetonitrile/methanol = 50:50. The peak alignment and identification were performed using LabSolutions Insight for LC/MS (Shimadzu Co.). The quantitative values were calculated by the internal standard method based on the peak area values for bile acids and their appropriate internal standards, and normalized against the sample weights (nmol/mg small-intestinal content).

### 2.6. FITC-Dextran Assay

Mice were administered fluorescein isothiocyanate (FITC) dextran (molecular weight, 4000; Sigma-Aldrich) orally. Three hours later, the mice were sacrificed and blood was collected from the inferior vena cava. The serum concentration of FITC-dextran was measured with a fluorometer (excitation, 485 nm; emission, 528 nm) as previously described [17].

### 2.7. Statistical Analysis

All values were expressed as means ± SD. The significance of differences between two animal groups was analyzed by the Mann–Whitney *U*-test. For analyses of gut microbiota, statistical significance was determined by Welch’s t-test with Benjamini–Hochberg correlation. Differences were considered to be significant at *p* < 0.05.

## 3. Results

### 3.1. Effect of a High-Fat Diet on Body Weight and Physiology in the Small Intestine and Liver

**Figure 1:**
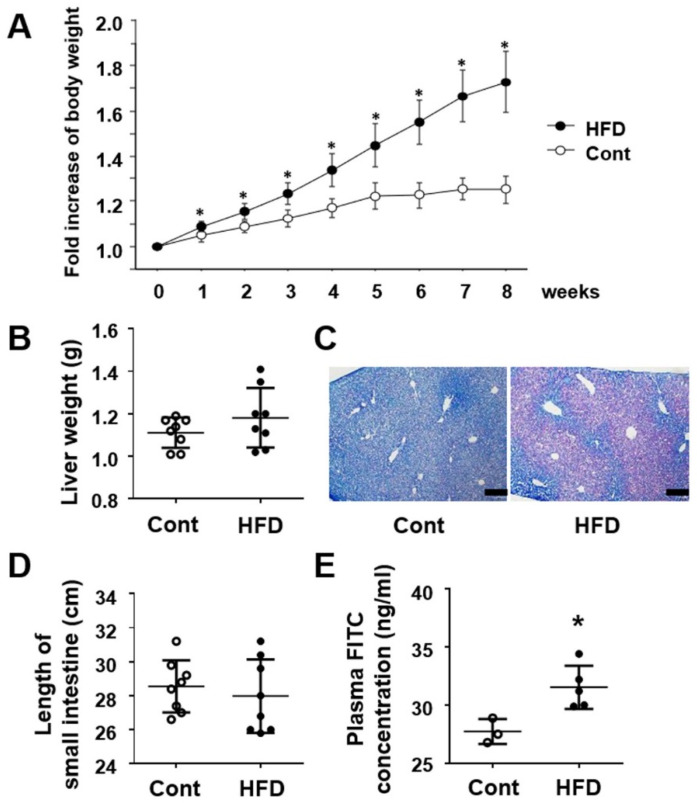
Effect of a HFD on body weight, liver tissues and small-intestinal tissues. (**A**) Change in body weight (each group, *n* = 8). (**B**) Weight of the liver (each group, *n* = 8). (**C**) Nile blue staining of the liver tissues. Bar = 200 μm. (**D**) Length of the small intestine (each group, *n* = 8). (**E**) Plasma concentration of absorbed FITC-dextran (Cont, *n* = 3; HFD, *n* = 5). Results are expressed as the mean ± SD. * *p* < 0.05 vs. control group. Cont, control; HFD, high-fat diet.

Body weight increased naturally in both the control and HFD-treated groups. The percentage increase in body weight was significantly greater in HFD-treated groups from one week after the start of the experiment (Figure 1A). The weight of the liver tended to increase in the HFD group but no significant difference was evident during the experimental period (Figure 1B). However, pathological examination of the liver tissues revealed apparent accumulation of fat droplets in the liver of mice fed the HFD (Figure 1C). For the small intestine, although no difference in length was evident (Figure 1D), the serum level of FITC was significantly higher in the HFD group than in the controls (Figure 1E), suggesting that intestinal permeability had accelerated in the HFD-fed mice.

### 3.2. Effect of a High-Fat Diet on Expression of Tight Junction Proteins in the Small Intestine

**Figure 2:**
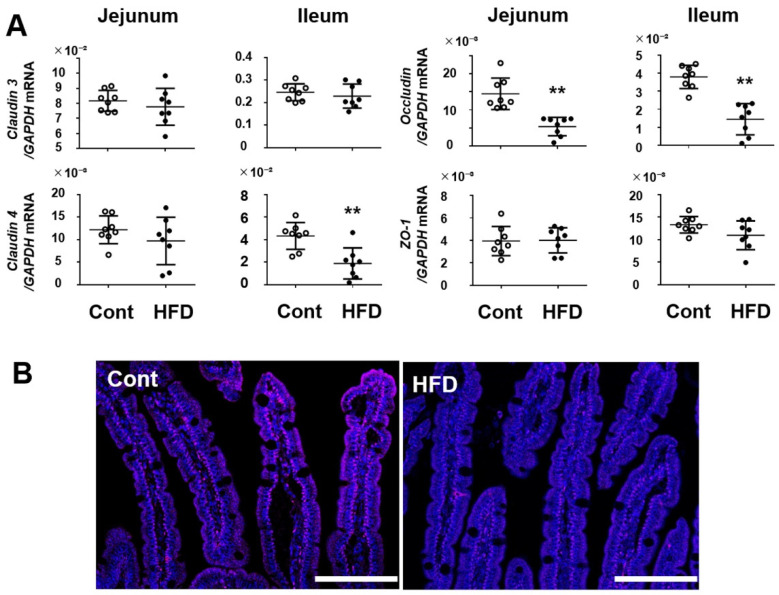
(**A**) Effect of a HFD on mRNA expression of tight junction molecules in mouse small-intestinal tissues. (**B**) Immunostaining of occludin in the jejunal mucosa. Red signal is the immunoreactivity for occludin. Bar = 100 μm. Results are expressed as the mean ± SD. ** *p* < 0.01 vs. control group. Cont, control (*n* = 8); HFD, high-fat diet (*n* = 8).

We next investigated the mRNA expression of tight junction proteins, which play a pivotal role in the barrier function of the small-intestinal mucosa. As shown in Figure 2A, the expression of claudin 4 was significantly decreased in the ileum but not in the jejunum of the mice fed the HFD. The expression of neither claudin 3 nor ZO-1 differed between the control and the HFD groups. However, the expression of occludin was significantly decreased in both the jejunum and ileum of HFD-fed mice. Indeed, immunohistochemistry showed that occludin expression was apparently decreased in the small-intestinal epithelium in the HFD-fed mice relative to the controls (Figure 2B).

### 3.3. Effect of a High-Fat Diet on Gut Flora in the Small Intestine

**Figure 3:**
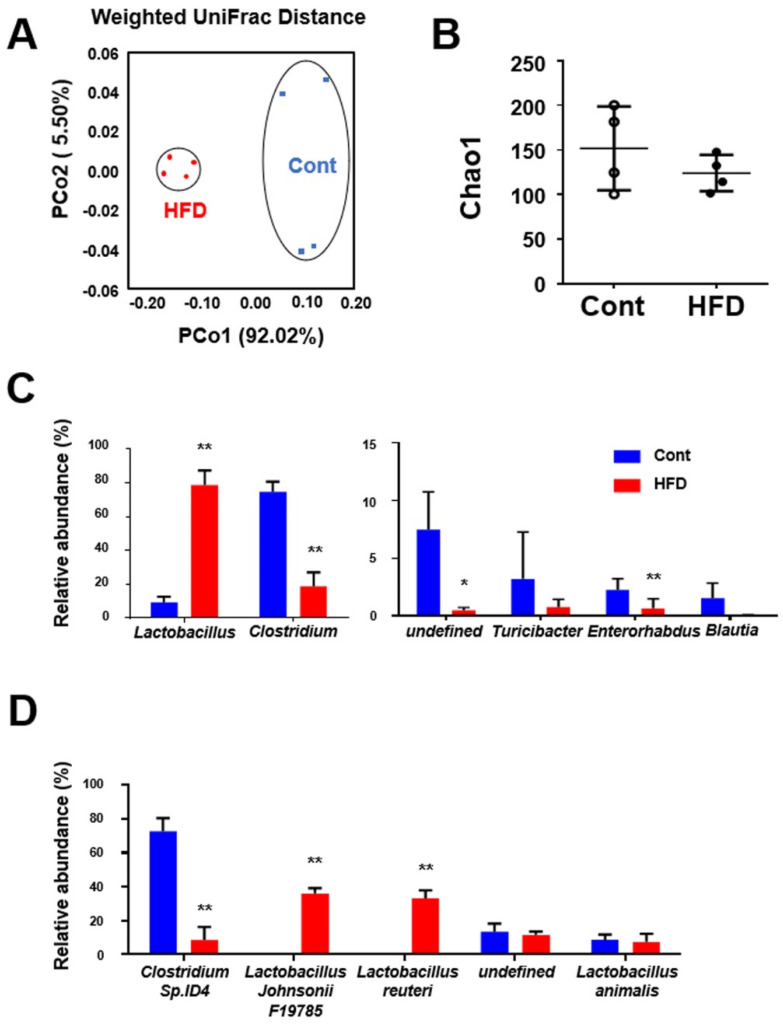
Effect of a HFD on gut microbiota in the small intestine. (**A**) Weighted UniFrac principal coordinate analyses (PCoA) showing clustered communities of small-intestinal microbiota in the experimental mice. PCo1 and PCo2 describe the indicated percentage of variation on the x-axis and y-axis, respectively. (**B**) Chao1 indicating α-diversity of the gut microbiota. The relative abundance of small-intestinal bacteria at (**C**) the genus and (**D**) the species levels. Results are expressed as the mean ± SD. * *p* < 0.05; ** *p* < 0.01 vs. control group. Cont, control (*n* = 4); HFD, high-fat diet (*n* = 4).

Since HFD is likely to cause dysbiosis [7] we analyzed the alteration of the gut microbiome profile in the experimental mice. Weighted UniFrac-based principal coordinate analysis (PCoA) revealed the difference in the gut microbiota structure between the control and the HFD-fed mice (Figure 3A). Chao1 indices for α-diversity assessment did not differ between the two groups (Figure 3B).

Furthermore, we investigated the genus profile of the gut microbiome in the experimental mice (Figure 3C). Among the major genera (relative abundance ≥1%), *Lactobacillus* was markedly more abundant in the HFD-fed mice than in the controls (*p* < 0.01; control, 9.21 ± 2.78%; HFD, 78.67 ± 7.54%). In contrast, *Clostridium* was drastically less abundant in HFD-fed mice (*p* < 0.01; control, 74.62 ± 5.27%; HFD, 18.61 ± 7.25%). In addition, *Enterorhabdus* was significantly decreased in HFD-treated mice, and *Turicibacter* and *Blautia* tended to be decreased in those mice. Data for genera where the relative abundance was <1% are presented in Supplementary Figure S1.

We also analyzed the species profile of the gut microbiome in the small-intestinal contents (Figure 3D). Among the major species (relative abundance ≥5%), *Clostridium* sp. ID4 (currently named *Fecalibaculum rodentium*) was markedly less abundant in HFD-fed mice than in the controls (*p* < 0.01). On the other hand, *Lactobacillus johnsonii* and *Lactobacillus reuteri* were specifically more abundant in the HFD-fed mice (*p* < 0.01). The data for species where the relative abundance was <5% are presented in Supplementary Figure S2.

### 3.4. Effect of a High-Fat Diet on the Luminal Environment of the Small Intestine

**Figure 4:**
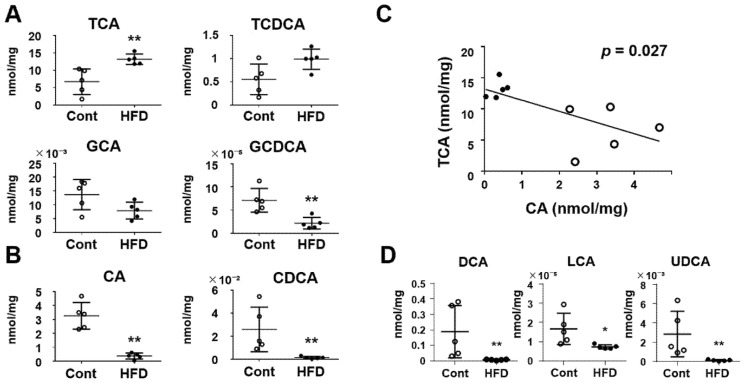
Effect of a HFD on concentrations of bile acids in the small-intestinal contents. (**A**) Conjugated primary bile acids. (**B**) Deconjugated primary bile acids. (**C**) Correlation between TCA and CA. *p* < 0.05 by linear regression analysis. (**D**) Secondary bile acids. Results are expressed as the mean ± SD. * *p* < 0.05; ** *p* < 0.01 vs. control group. Cont, control (*n* = 5); HFD, high-fat diet (*n* = 5).

We then analyzed the luminal contents of the small intestine of the experimental mice. The pH of the small-intestinal content was significantly lower in HFD-fed mice (6.70 ± 0.22) than in the controls (7.13 ± 0.14) (*p* < 0.01). The gut microbiome plays a pivotal role in the deconjugation of conjugated bile acids [18], and moreover, conjugated bile acids have the ability to form micelles with lipids and play a crucial role in the absorption of lipids in the small intestine [19]. Therefore, we analyzed the concentrations of various conjugated bile acids and deconjugated primary/secondary bile acids in the intestinal contents (Figure 4). Among the conjugated bile acids (Figure 4A), the basic concentrations of taurine-conjugated bile acids including TCA and TCDCA were much higher than those of glycine-conjugated bile acids including GCA and GCDCA, in both the controls and the HFD-fed mice. The level of TCA was significantly higher in the HFD-fed mice than that in the controls. On the other hand, the level of CA, which is a deconjugated primary bile acid, was significantly lower in the HFD group (Figure 4B). These finding suggest that the production of TCA is increased whereas deconjugation of taurine for CA is inhibited in mice fed an HFD. With regard to CDCA, both glycine-conjugated and -deconjugated CDCA were significantly decreased in HFD-fed mice (Figure 4A,B), suggesting that the production of GCDCA itself is decreased in those mice. When analyzing the correlation between TCA and CA in the small-intestinal contents, the levels of TCA and CA were negatively correlated (Figure 4C; *p* < 0.05). Furthermore, we evaluated the levels of secondary bile acids in the small-intestinal contents. All of the three secondary bile acids, DCA, LCA and UDCA, were significantly decreased in the HFD-fed mice relative to the controls (Figure 4D).

### 3.5. Effect of a High-Fat Diet on Expression of Antimicrobial Peptides, Cytokines and LPS in the Small Intestine

**Figure 5:**
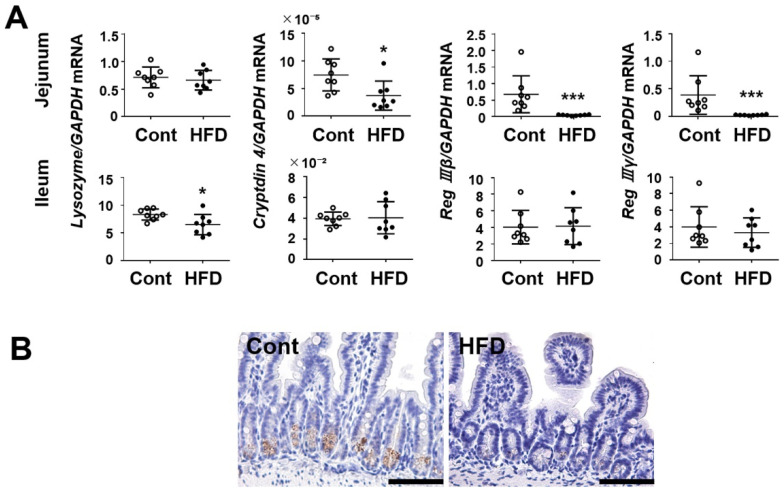
(**A**) Effect of a HFD on mRNA expression of antimicrobial peptides in mouse small-intestinal tissues. (**B**) Immunostaining of lysozyme in the ileum. Bar = 100 μm. Results are expressed as the mean ± SD. * *p* < 0.05; *** *p* < 0.001 vs. control group. Cont, control (*n* = 8); HFD, high-fat diet (*n* = 8).

We investigated the luminal environment in terms of inflammation-associated molecules. The expression of anti-microbial peptides such as cryptdin 4 was significantly decreased in the jejunum of HFD-fed mice (Figure 5). On the other hand, lysozyme expression was decreased in the ileum of those mice. Interestingly, the expression of Reg III β/α was drastically suppressed in the jejunum of HFD-fed mice relative to the controls (*p* < 0.001). As shown in Figure 5B, the immunoreactivity of lysozymes was localized at Paneth cells in the small intestine and its intensity was apparently reduced in HFD-fed mice.

**Figure 6:**
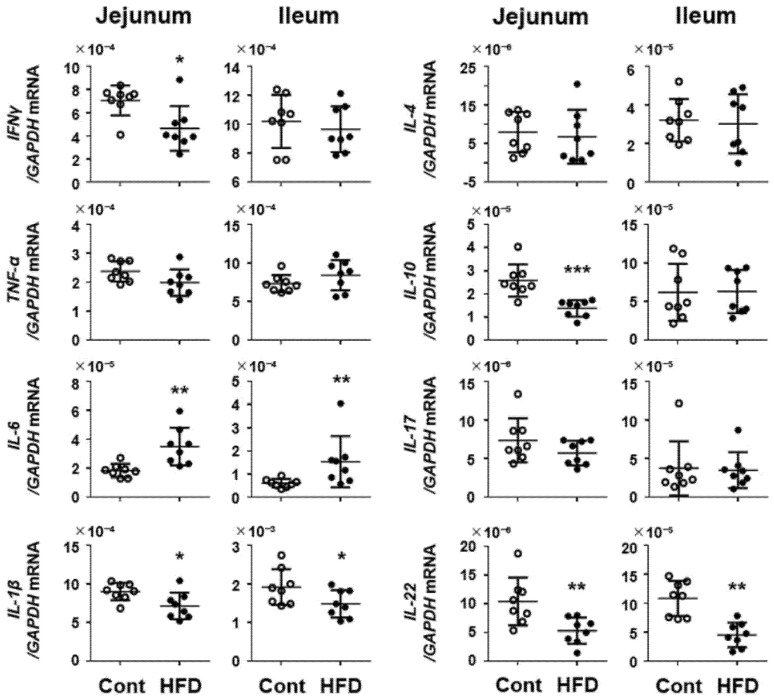
Effect of a HFD on mRNA expression of cytokines in mouse small-intestinal tissues. Results are expressed as the mean ± SD. * *p* < 0.05; ** *p* < 0.01 vs. control group. Cont, control (*n* = 8); HFD, high-fat diet (*n* = 8).

Figure 6 shows the profile of cytokine expression in the small intestine of the experimental mice. In mice fed with HFD, the expression of IL-6 was significantly elevated throughout the small intestine, whereas that of IL-1β was decreased. Of note, expression of the anti-inflammatory cytokine IL-10 was significantly decreased in the HFD group, and the expression of IL-22, which is crucial for mucosal innate immunity, was significantly decreased throughout the small intestine. Besides this, we investigated the behavior of lymphocytes in the small intestinal mucosa. As shown in Supplementary Figure S4, the population of CD3-positive lymphocytes in the jejunum did not differ between controls and HFD groups.

**Figure 7:**
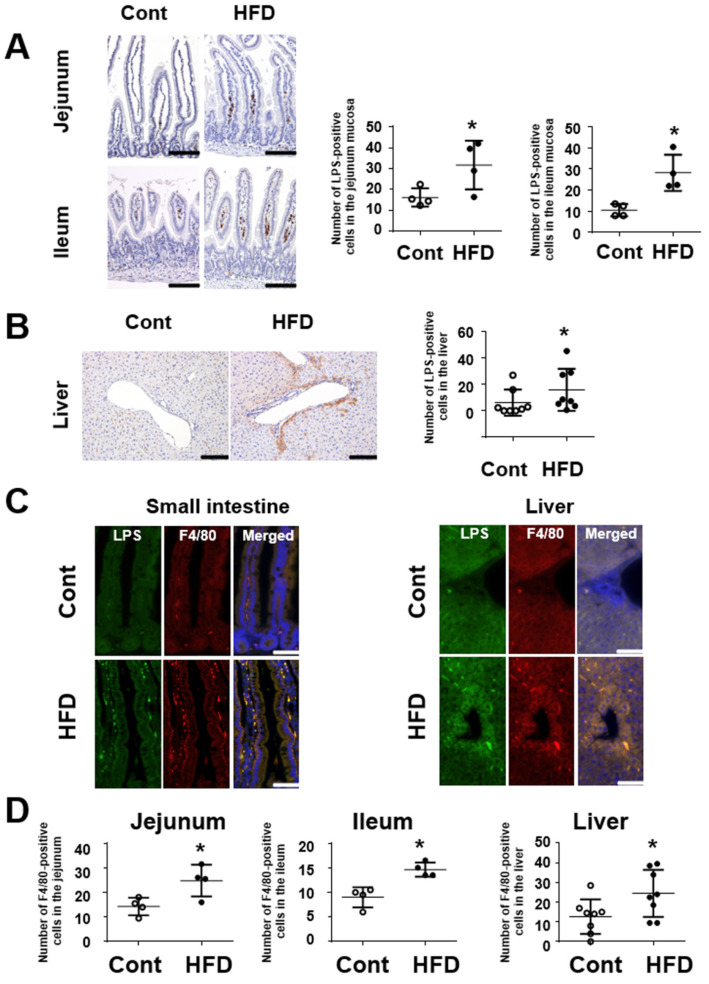
Effect of a HFD on immunoreactivity of LPS in the small intestine and liver in mice. (**A**) Images showing immunostaining of LPS in the small intestine. Graphs showing the number of LPS-positive cells in the small-intestinal mucosa (each group, *n* = 4). Bar = 100 μm. (**B**) Images showing immunostaining of LPS in the liver. Graphs showing the number of LPS-positive cells in the liver (each group, *n* = 8). Bar = 100 μm. (**C**) Immunohistochemical double staining for LPS (green) and F4/80 (red) in the small intestine and the liver. Bar = 50 μm. (**D**) Number of F4/80-positive cells in the small intestine and the liver. Results are expressed as the mean ± SD. * *p* < 0.05 vs. control group. Cont, control; HFD, high-fat diet.

To investigate the invasion of harmful antigens into the small-intestinal mucosa and liver tissues, we examined the immunoreactivity of LPS in those organs using immunohistochemistry. Immunoreactivity for LPS was detected mainly in the lamina propria of the small-intestinal mucosa (Figure 7A). The number of LPS-positive cells was significantly increased in the HFD group relative to the controls. In liver tissues, LPS immunoreactivity was observed to mainly surround interlobular veins. (Figure 7B). To clarify which cells were positive for LPS immunoreactivity, we performed double-immunostaining using antibodies against LPS and the macrophage marker F4/80. As shown in Figure 7C, some signals for LPS were colocalized in F4/80-positive cells of not only the small-intestinal mucosa but also the liver. The number of F4/80 cells was significantly increased in the HFD group relative to the controls in both the small intestine and the liver (Figure 7D).

## 4. Discussion

It is evident that ingestion of a HFD causes not only steatohepatitis but also metabolic syndrome, although the underlying pathogenesis has not been fully clarified [20]. Indeed, we have clearly shown in the present study that body weight was significantly increased in HFD-fed mice relative to controls, and marked accumulation of fat drops was observed in the former. Recent evidence suggests that disruption of the intestinal mucosa barrier is a key trigger for the development of HFD-associated steatohepatitis [21]. The intestinal mucosa barrier protects the host from invasion by pathogens or harmful antigens, and therefore, its disruption (so called “leaky gut”) facilitates their invasion, promoting inflammation in not only the gastrointestinal tract but also the liver [22]. As we have shown in this study, the permeability of the gastrointestinal tract was significantly increased in mice fed the HFD, and this was likely associated with the development of hepatic steatosis. The intestinal mucosal epithelium is sealed by tight junction proteins, which maintain the barrier function by regulating the permeability of the intestinal mucosa [23]. Our investigation of the expression level of tight junction proteins revealed that *occludin* was significantly decreased throughout the small intestine in the HFD-fed mice. This may have been at least partly associated with the increased permeability of the gastrointestinal tract in those mice.

The luminal environment is a crucial factor that affects intestinal barrier function. HFD-fed mice are a well-established model for studying the pathophysiology of metabolic syndrome. For instance, some papers showed the decrease of *Lactobacillus* spp. and the increase of *Clostridium* spp. in the colon of HFD-fed mice [7,20] although conflicting data are also reported [24,25]. On the other hand, the information on the small-intestinal luminal environment in this model is limited. In this connection, we examined the small-intestinal luminal contents in terms of the gut microbiome profile and showed that this profile in HFD-fed mice was quite different from that in the controls. Interestingly, in control mice, most of the gut microbiome comprised *Clostridium* (approximately 70%) and *Lactobacillus* (approximately 10%), whereas this situation was completely reversed (*Clostridium*, approximately 20%; *Lactobacillus*, approximately 75%) in HFD-fed mice. These findings suggest that a HFD drastically affects the profile of the gut flora in the small intestine. Furthermore, we found that the pH of the small-intestinal luminal contents was significantly decreased in mice fed the HFD. Since *Lactobacillus* plays a pivotal role in the promotion of glycolysis [26], the resulting acid products of glycolysis might contribute to acidification of the small-intestinal luminal contents. On the other hand, it is an interesting question whether the correction of dysbiosis recovers the pathophysiology induced by the treatment with HFD. In this regard, a few papers have demonstrated that probiotic treatment prevents HFD-associated steatohepatitis in rodent models [27,28]. This may suggest that some gut microbiomes are likely to improve the HFD-associated pathophysiology although there is no direct evidence as to whether the improvement of dysbiosis really recovers the complete HFD-associated pathophysiology.

The number of gut microbiome species in the small intestine is much smaller than that in the colon [29]. It is well known that one of crucial roles of the colonic gut microbiome is fermentation of diet-derived fibers, producing short-chain fatty acids [30]. On the other hand, in the small intestine, the gut microbiome plays a pivotal role in the transformation of bile acids [18]. Bile acids, especially conjugated ones, have the ability to form micelles with lipids and play a crucial role in the absorption of lipids in the small intestine [19]. Therefore, we investigated the profile of bile acids in the small-intestinal luminal contents in experimental mice and found that TCA and CA are main bile acids contained in the small-intestinal lumen. Interestingly, we found that deconjugation of taurine was strongly inhibited in mice fed a HFD, resulting in an increase of conjugated primary bile acids. Deconjugation of bile acids is promoted by the activation of bile salt hydrase, and all major bacteria including *Clostridium* and *Bacteroides* are known to have this enzyme activity [31,32,33]. Accordingly, it is tempting to speculate that the marked decrease of *Clostridium* sp. *ID4* may be related to inhibition of bile acid deconjugation in HFD-fed mice, although other possibilities cannot be excluded. *Clostridium* sp. *ID4* has now been re-classified into the phylum *Firmicutes* and named *Faecalibaculum rodentium* [34], possibly playing an anti-inflammatory role in the intestinal mucosa [35]. In this context, a decrease of *Clostridium* sp. *ID4* might be disadvantageous for not only bile acid transformation but also mucosal integrity. On the other hand, since conjugated bile acid can easily be reabsorbed and is likely to promote the absorption of lipid, any increase of conjugated bile acid might accelerate the accumulation of lipid in the liver. In the small intestine of HFD-fed mice, we found that not only deconjugated primary bile acids but also secondary ones were decreased. Secondary bile acids are produced from deconjugated primary bile acids by dehydration [36]. Therefore, the decreased level of secondary bile acids may reflect the decreased level of deconjugated primary bile acids in HFD-fed mice.

As we have demonstrated in this study, intake of a HFD greatly alters the gut microbiome and luminal contents of the small intestine. Furthermore, we have also found that the expression of antimicrobial peptides such lysozyme and Reg IIIβ/γ is decreased in the small-intestinal mucosa. These may negatively impact the ability of the mucosal barrier to protect the small intestine from pathogen invasion. Furthermore, since the antimicrobial peptides examined are produced in Paneth cells [37,38], those findings may reflect the disturbance of Paneth cells by HFD treatment. Interestingly, the production of an antimicrobial peptide is largely affected by the diet [39], and moreover, a HFD is likely to suppress the expression of antimicrobial peptides including lysozymes and Reg IIIβ/γ in the small intestine [40]. At present, the mechanisms of expression of antimicrobial peptides are not fully understood. However, it is interesting to note that the expression of antimicrobial peptides is very weak in germ-free mice whereas it is markedly increased by transplantation of commensal bacteria [41]. This suggests that the presence of commensal bacteria may be essential for the expression of antimicrobial peptides. Although it may be impossible to identify the bacterial strains responsible for the expression of antimicrobial peptides, some candidate strains may be part of the reduced microbiome in mice fed an HFD.

In this study, we also investigated the immune system in the small-intestinal mucosa of HFD-fed mice, because low-level inflammation in the small intestine may underlie the pathophysiology of gut-liver axis disorders [42]. LPS immunoreactivity was augmented in not only the small-intestinal mucosa but also the liver tissues of mice fed a HFD, in agreement with previous reports [5]. This may suggest that invasion of pathogens through the mucosal barrier is accelerated, being compatible with an increase of intestinal mucosa permeability. Among the alterations of cytokine expression in the small intestine of HFD-fed mice, expression of the proinflammatory cytokine *IL-6* was found to be significantly increased. This suggests that HFD-fed mice may have low-level inflammation linked to LPS infiltration in the small-intestinal mucosa. On the other hand, the expression of IL-22, which plays a pivotal role in innate immunity, was significantly decreased in the small intestine of HFD-fed mice. Interestingly, antimicrobial peptides including lysozyme and Reg IIIβ/γ, which function in the first-line defense of the intestinal mucosa from pathogens [43], are target molecules for IL-22 signaling in innate immunity [44]. Thus, suppression of the IL-22/antimicrobial peptide axis may be at least partly associated with low-level inflammation in the small intestine of HFD-fed mice.

In summary, we have shown that intake of a HFD in mice alters the small-intestinal gut flora and bile acid profile, accompanied by acceleration of gut permeability and a decrease of TJ protein expression in the small-intestinal mucosa. Moreover, we have demonstrated that the expression of IL-22/antimicrobial peptides is significantly decreased in the small intestine of these mice. Subsequently, infiltration of LPS was increased in not only the small-intestinal mucosa but also the liver, possibly contributing to the development of chronic low-level inflammation in the small intestine and steatohepatitis. The various interrelationships among HFD-induced alterations of the gut flora, bile metabolism, antimicrobial peptides and mucosal permeability in the small intestine still remain to be clarified. However, the present findings at least suggest that HFD-induced alteration of the luminal environment is closely associated with low-level inflammation in the small intestine, affecting the gut-liver axis by disturbing the small-intestinal mucosal integrity.

## Data Availability

The data presented in this study are available on request from the corresponding author.

## Supplementary Materials

The following are available online at https://www.mdpi.com/article/10.3390/cells10113168/s1, Supplementary Figure S1: Effect of a HFD on the relative abundance of small-intestinal bacteria at the genus level., Supplementary Figure S2: Effect of a HFD on the relative abundance of small-intestinal bacteria at the species level., Supplementary Methods S1: Illumina library generation and DNA sequencing.
