# Supplementary material for: Effect of a High-Fat Diet on the Small-Intestinal Environment and Mucosal Integrity in the Gut-Liver Axis
Source: Cells. 2021 Nov 14;10(11):3168. doi: 10.3390/cells10113168 (PMC8622719; doi:10.3390/cells10113168)
Supplement: Supplementary file 1 [file cells-10-03168-s001.zip › Spplementary Table S1 revise.pdf]

Supplementary Table S1. The compositions of normal and high-fat diet.

**Ingredient (g/kg)**	**Normal diet**	**High-fat diet**
Casein	189.6	258.5
L-Cystine	2.8	3.9
Corn starch	479.8	0
Maltodextrin	118.5	161.5
Sucrose	65.2	88.9
Cellulose	47.4	64.6
Soybean oil	23.7	32.3
Lard	19.0	316.6
Mineral mix S10026	9.5	12.9
Dicalcium phosphate	12.3	16.8
Calcium carbonate	5.2	7.1
Potassium citrate	15.6	21.3
Vitamin mix V10001	9.5	12.9
Choline bitartrate	1.9	2.6
Total	1000	1000
**Energy (% kcal)**	**Normal diet**	**High-fat diet**
Protein	20	20
Carbohydrate	70	20
Fat	10	60
Total	100	100
