# Supplementary material for: Effect of a High-Fat Diet on the Small-Intestinal Environment and Mucosal Integrity in the Gut-Liver Axis
Source: Cells. 2021 Nov 14;10(11):3168. doi: 10.3390/cells10113168 (PMC8622719; doi:10.3390/cells10113168)
Supplement: Supplementary file 1 [file cells-10-03168-s001.zip › Spplementary Table S2 revise.pdf]

Supplementary Table S2. Mouse Primers for real-time reverse transcription-polymerase chain reaction analysis.

Genes	Direction	Sequences
Lysozyme	Forward	5'-CAAGATCTAAGAATGCCTGTG-3’
	Reverse	5’-TTCCGAATATACTGGGACAG-3’
Cryptdin 4	Forward	5’-GTCCAGGCTGATCCTATCCA-3’
	Reverse	5’-GGGGCAGCAGTACAAAAATC-3’
Reg IIIβ	Forward	5’-TCCCAGGCTTATGGCTCCTA-3’
	Reverse	5’-GCAGGCCAGTTCTGCATCA-3’
Reg IIIγ	Forward	5’-TTCCTGTCCTCCATGATCAAAA-3’
	Reverse	5’-CATCCACCTCTGTTGGGTTCA-3’
Claudin 3	Forward	5’-CCAACTGCGTACAAGACGAG-3’
	Reverse	5’-TCTTGGTGGGTGCATACTTG-3’
Claudin 4	Forward	5’-GGAGGGCCTCTGGATGAACT-3’
	Reverse	5’-GATGCTGATGACCATAAGGGC-3’
Occludin	Forward	5’-TGAAAGTCCACCTCCTTACAGA-3’
	Reverse	5’-CCGGATAAAAAGAGTACGCTGG-3’
ZO-1	Forward	5’-GAGCTACGCTTGCCACACTGT-3’
	Reverse	5’-TCGGATCTCCAGGAAGACACTT-3’
IFN-γ	Forward	5’-GCATCTTGGCTTTGCAGCT-3’
	Reverse	5’-CCTTTTTCGCCTTGCTGTTG-3’
TNF-α	Forward	5’-GGTGCCTATGTCTCAGCCTCTT-3’
	Reverse	5’-GCCATAGAACTGATGAGAGGGAG-3’
IL-1β	Forward	5’-TCCAGGATGAGGACATGAGCAC-3’
	Reverse	5’-GAACGTCACACACCAGCAGGTTA-3’
IL-4	Forward	5’-GAATGTACCAGGAGCCATATC-3’
	Reverse	5’-CTCAGTACTACGAGTAATCCA-3’
IL-6	Forward	5’-CCAGTTGCCTTCTTGGGACT-3’
	Reverse	5’-GGTCTGTTGGGAGTGGTATCC-3’
IL-10	Forward	5’-TGGACAACATACTGCTAACCG-3’
	Reverse	5’-GGATCATTTCCGATAAGGCT-3’
IL-17A	Forward	5’-GACTCTCCACCGCAATG-3’
	Reverse	5’-CGGGTCTCTGTTTAGGCT-3’
IL-22	Forward	5’-TCCGAGGAGTCAGTGCTAAA-3’
	Reverse	5’-AGAACGTCTTCCAGGGTGAA-3’
GAPDH	Forward	5’-GGAGAAACCTGCCAAGTATG-3’
	Reverse	5’-TGGGAGTTGCTGTTGAAGTC-3’

IL, interleukin; *IFN-γ*, interferon-γ; *TNF-α*, tumor necrosis factor-α; *ZO-1*, Zona occludens-1.
