# Supplementary material for: Effect of a High-Fat Diet on the Small-Intestinal Environment and Mucosal Integrity in the Gut-Liver Axis
Source: Cells. 2021 Nov 14;10(11):3168. doi: 10.3390/cells10113168 (PMC8622719; doi:10.3390/cells10113168)
Supplement: Supplementary file 1 [file cells-10-03168-s001.zip › Supplementary Methods S1 revise.pdf]

Supplementary Methods S1

Illumina library generation and DNA sequencing

Extraction of bacterial DNA was performed as described previously (1, 2). The small-intestinal contents (20 mg) were suspended in 1.0 mL of PBS and centrifuged (14000 × *g*) to remove possible PCR inhibitors three times. The pellets were resuspended in a solution containing 450 µL of extraction buffer (100 mM Tris-HCl, 40 mM EDTA; pH 9.0) and 50 µL of 10% sodium dodecyl sulfate. Three hundred milligrams of glass beads (diameter, 0.1 mm) and 500 µL of buffer-saturated phenol were added to the suspension, and the mixture was disrupted using a Micro Smash (4000 rpm, 10 seconds, TOMY SEIKO, Tokyo, Japan). After disruption, the mixture was incubated at 65 °C in a water bath for 10 min. Again, the mixture was disrupted and incubated at 65 °C for 10 min. After centrifugation at 20,000 × *g* for 10 min, 400 µl of the supernatant was collected. Subsequently, phenol-chloroform extractions were performed, and 250 µL of the supernatant was subjected to isopropanol precipitation. Finally, the DNA was suspended in 1 ml of Tris-EDTA buffer.

Analysis of 16S rDNA in the microbial community present in the small-intestinal contents was performed in accordance with a method described previously (3) with minor modifications. In brief, the V3-V4 region of 16S rDNA was amplified using a forward

primer (5′-TCGTCGGCAGCGTCAGATGTGTATAAGAGACAG CCTACGGGNGGCWGCAG-3′) and a reverse primer (5′-GTCTCGTGGG CTCGGAGATGTGTATAAGAGACAGGACTACHVGGGTATCTAATCC-3′), which were ligated with overhang Illumina adapter consensus sequences. The PCR was performed using the following program: 95 °C for 3 min, followed by 28 cycles consisting of 95 °C for 30 s, 55 °C for 30 s and 72 °C for 30 s. After 28 cycles, the reaction was completed with a final extension of 5 min at 72 °C on a Veriti thermal cycler (Thermo Fisher Scientific, Waltham, MA, USA). The amplicon was purified using AMPure XP magnetic beads (Beckman Coulter, Brea CA, USA). The Illumina Nextera XT Index kit (Illumina) with dual 8-base indices was used to allow for multiplexing. To incorporate two unique indices to the 16S amplicons, PCR reactions were performed. Cycling conditions consisted of 95 °C for 3 min, followed by 8 cycles of 95 °C for 30 s, 55 °C for 30 s and 72 °C for 30 s, followed by a final extension cycle of 72 °C for 5 min. After purification using AMPure XP beads, the purified barcoded library was quantified fluorometrically using a QuantiT PicoGreen ds DNA Assay Kit (Invitrogen, Paisley, UK). Libraries were then diluted to 4 nM using 10 mM Tris-HCl (pH 8.5), followed by pooling of the same volume for multiplex sequencing. The multiplexed library pool (10 pM) was spiked with 40% PhiX control DNA (10 pM) to improve base calling during sequencing.

Sequencing was conducted using a 2 × 250-bp paired-end run on a MiSeq platform with MiSeq Reagent Kit v2 chemistry (Illumina).

Demultiplexing and removal of indices were performed using the MiSeq Reporter software (Illumina). Subsequently, sequence files were exported from the MiSeq Reporter software as a further step. Filtering out of low-quality sequences, removal of chimera sequences, construction of operational taxonomic units (OTUs), and taxonomy assignment were conducted using the Quantitative Insights Into Microbial Ecology (QIIME) pipeline (http://qiime.org/) (4). In brief, 30000 raw reads were randomly obtained from the sequence files for each sample and merged by fastq-join with the default setting. Consequently, sequence reads with an average quality value of <25 were removed, and then chimera-checked. Five thousand high-quality sequence reads were randomly obtained for each sample, and OTUs for total high-quality reads were constructed by clustering with a 97% identity threshold. The representative reads of each OTU were then assigned to the 16S rRNA gene database using UCLUST with ≥97% identity. Comparison of each taxon in the gut microbiota was conducted at the genus and species level. Beta diversity was estimated by computing the weighted UniFrac distance between samples, a phylogenic tree-based metric (5). To compare the differences in the overall bacterial gut microbiota structure, principal co-ordinate analysis was applied to

reduce the dimensionality of the resulting distance matrix. The Chao 1 index was calculated to investigate the alpha diversity of microbiota in the samples.

References

1. Fukui H, Oshima T, Tanaka Y, Oikawa Y, Makizaki Y, Ohno H, Tomita T, Watari J, Miwa H. Effect of probiotic Bifidobacterium bifidum G9-1 on the relationship between gut microbiota profile and stress sensitivity in maternally separated rats. Sci Rep. 2018;8:12384.
2. Matsuki, T.; Watanabe, K.; Fujimoto, J.; Kado, Y.; Takada, T.; Matsumoto, K.; Tanaka, R. Quantitative PCR with 16S rRNA-gene-targeted species-specific primers for analysis of human intestinal bifidobacteria. Appl. Environ. Microbiol. 2004, 70, 167–173.
3. Kawahara, T.; Makizaki, Y.; Oikawa, Y.; Tanaka, Y.; Maeda, A.; Shimakawa, M.; Komoto, S.; Moriguchi, K.; Ohno, H.; Taniguchi, K. Oral administration of Bifidobacterium bifidum G9-1 alleviates rotavirus gastroenteritis through regulation of intestinal homeostasis by inducing mucosal protective factors. PLoSONE 2017, 12, e0173979.

4. Caporaso, J.G.; Kuczynski, J.; Stombaugh, J.; Bittinger, K.; Bushman, F.D.; Costello, E.K.; Fierer, N.; Peña, A.G.; Goodrich, J.K.; Gordon, J.I.; et al. QIIME allows analysis of high-throughput community sequencing data. Nat. Methods 2010, 7, 335–336.

5. Lozupone, C. & Knight, R. UniFrac: a new phylogenetic method for comparing microbial communities. Appl Environ Microbiol 71, 8228–8235 (2005).
