# Supplementary figures and images for: Effect of a High-Fat Diet on the Small-Intestinal Environment and Mucosal Integrity in the Gut-Liver Axis
Source: Cells. 2021 Nov 14;10(11):3168. doi: 10.3390/cells10113168 (PMC8622719; doi:10.3390/cells10113168)

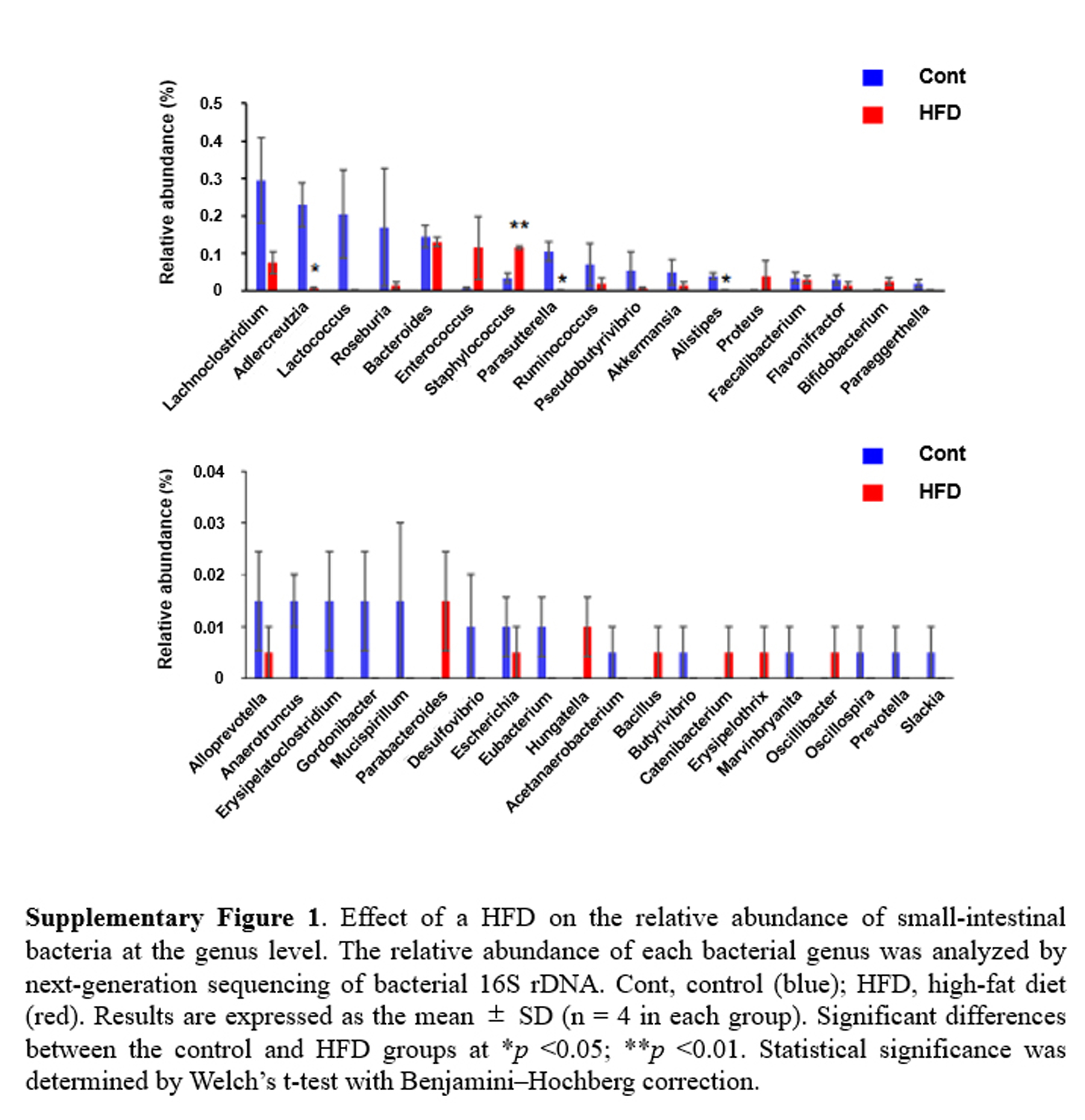

Supplement: Supplementary file 1 [file cells-10-03168-s001.zip › Supplementary Figure S1 revise.jpg]

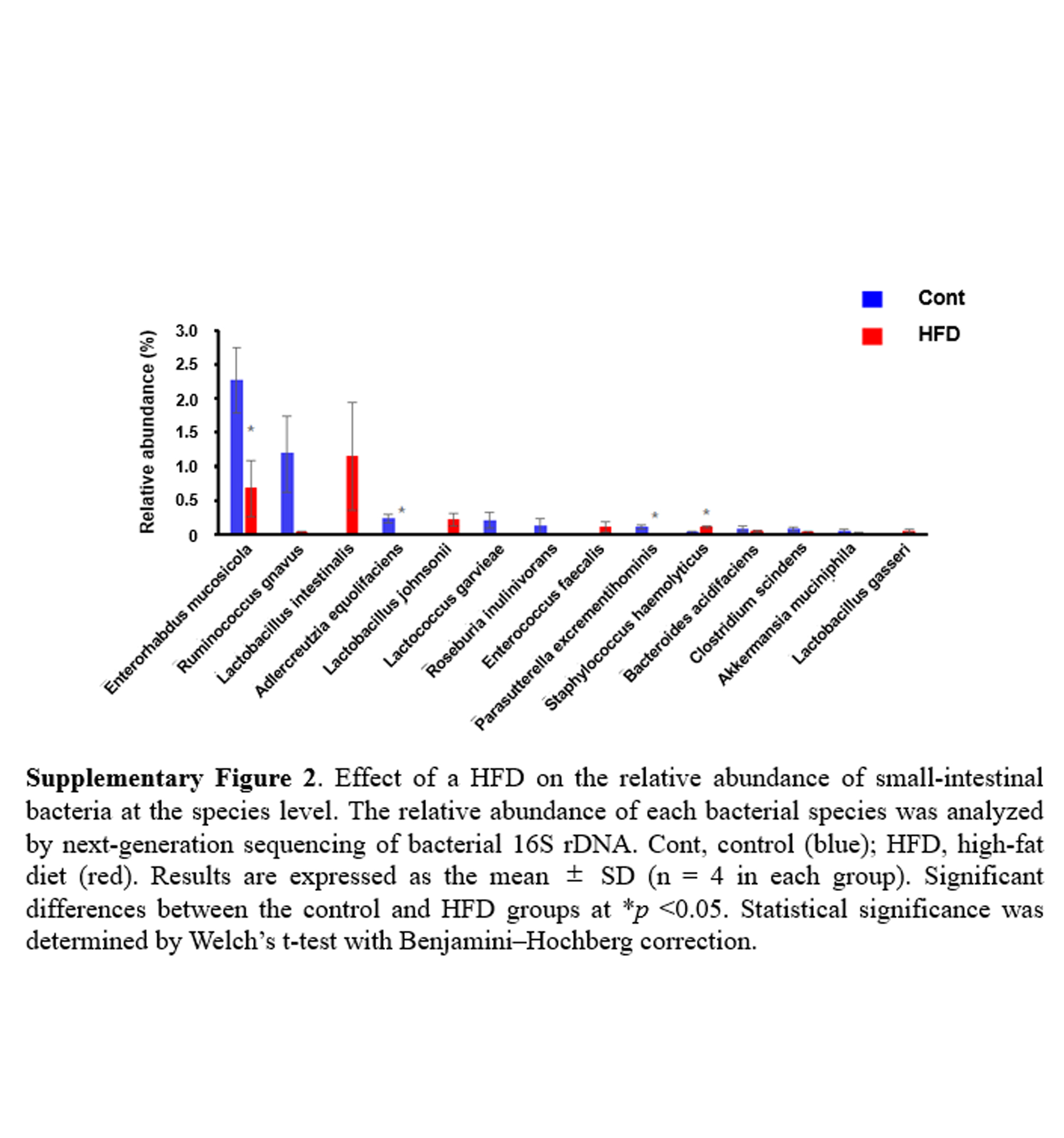

Supplement: Supplementary file 1 [file cells-10-03168-s001.zip › Supplementary Figure S2 revise.jpg]

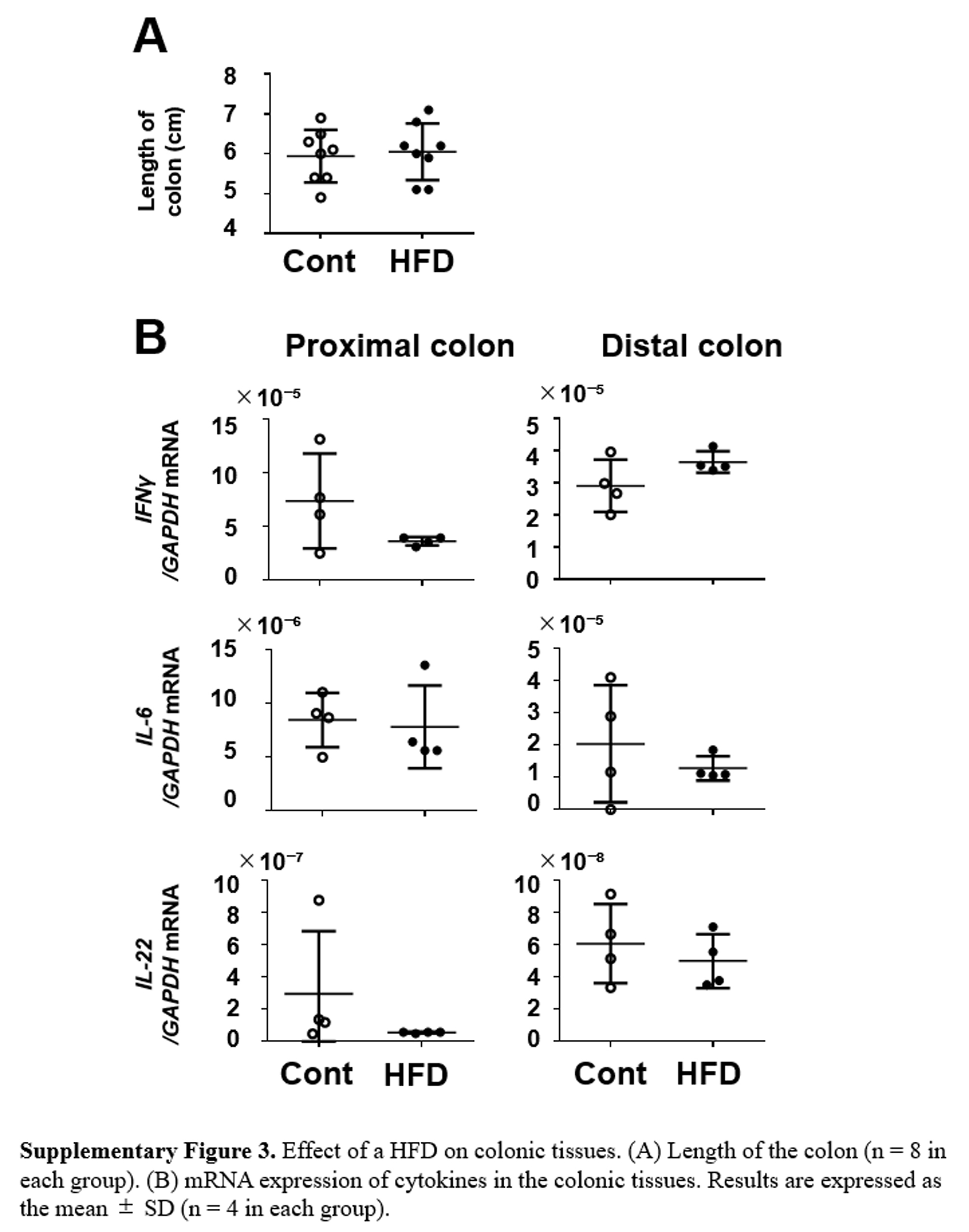

Supplement: Supplementary file 1 [file cells-10-03168-s001.zip › Supplementary Figure S3 revise.jpg]

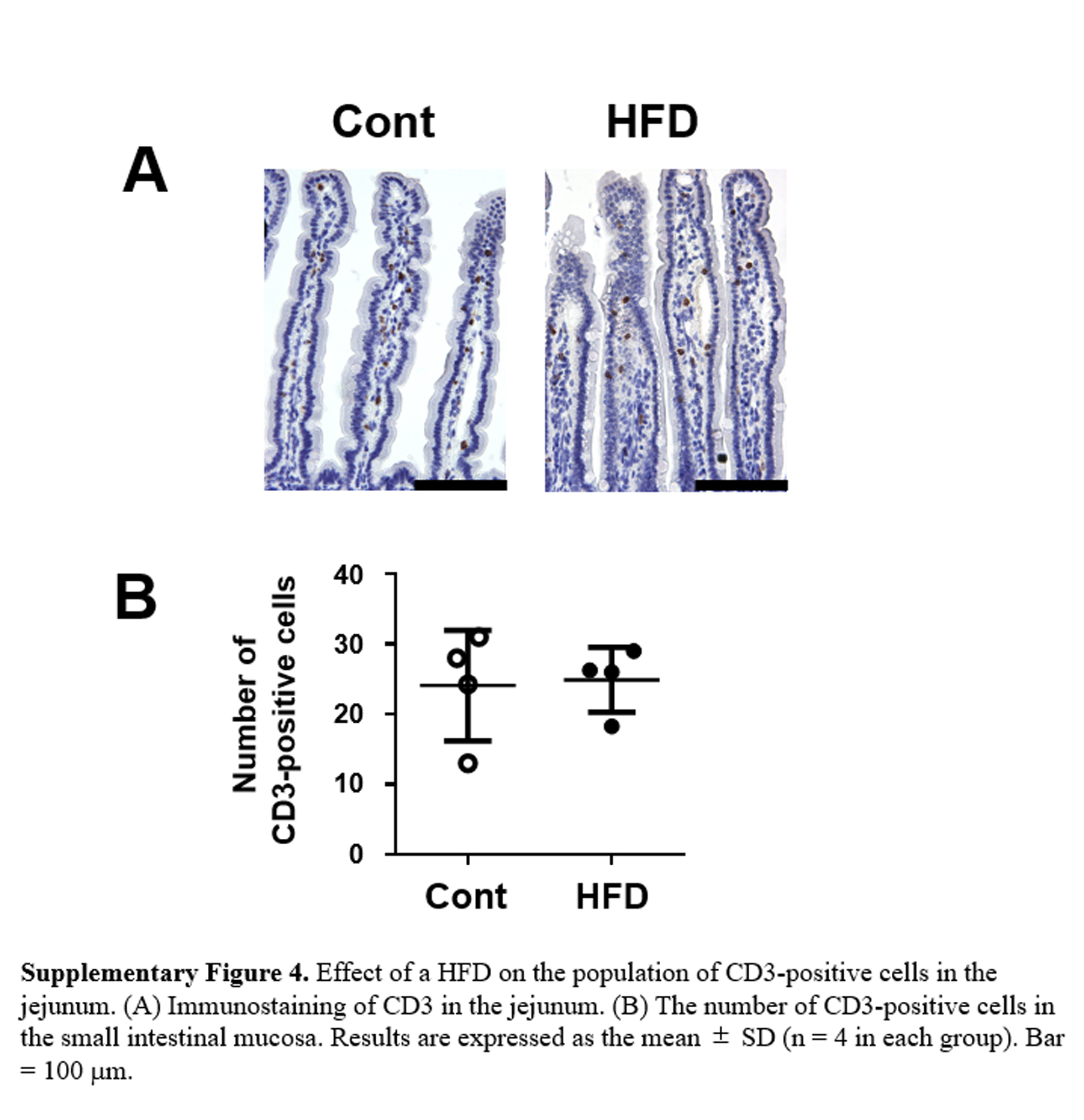

Supplement: Supplementary file 1 [file cells-10-03168-s001.zip › Supplementary Figure S4 revise.jpg]
